# Monitoring Sleep and Nightly Recovery with Wrist-Worn Wearables: Links to Training Load and Performance Adaptations

**DOI:** 10.3390/s25020533

**Published:** 2025-01-17

**Authors:** Olli-Pekka Nuuttila, Daniela Schäfer Olstad, Kaisu Martinmäki, Arja Uusitalo, Heikki Kyröläinen

**Affiliations:** 1 Faculty of Sport and Health Sciences, University of Jyväskylä, 40014 Jyväskylä, Finland; 2 UKK Institute for Health Promotion Research, 33500 Tampere, Finland; 3 Polar Electro Oy, 90440 Kempele, Finland; 4 Department of Sports and Exercise Medicine, Clinicum, University of Helsinki, 00014 Helsinki, Finland; 5 Helsinki Clinic for Sports and Exercise Medicine, Foundation for Sports and Exercise Medicine, 00550 Helsinki, Finland

**Keywords:** sleep monitoring, autonomic nervous system, nocturnal heart rate, nocturnal heart rate variability, overreaching

## Abstract

Previous studies on the effects of intensified training on sleep quality/quantity have been somewhat contradictory. Moreover, recreational athletes often track various sleep metrics, and those metrics’ actual connections to training adaptations are unknown. This study explored the effects of intensified training on sleep and nightly recovery along with their associations with training adaptations. A total of 24 participants (10 females) performed a 3-week baseline training period (BL), a 2-week overload period (OL), and a 1-week recovery period (REC), which were followed by test days (T1–T3). The endurance performance was assessed with a 3000 m running test. Throughout all of the periods, the nightly recovery information was monitored with a wrist-worn wearable, including sleep quantity and quality, heart rate (HR) and HR variability (HRV), and proprietary parameters combining several parameters and scaling the results individually. In addition, the perceived strain and muscle soreness were evaluated daily. The 3000 m running performance improved from T1 to T2 (−1.2 ± 1.7%, *p* = 0.006) and from T1 to T3 (−1.7 ± 1.2%, *p* = 0.002). The perceived strain and muscle soreness increased (*p* < 0.001) from the final week of the BL to the final week of the OL, but the subjective sleep quality and nightly recovery metrics remained unchanged. The OL average of the proprietary parameter, autonomic nervous system charge (“ANS charge”, combining the HR, HRV, and breathing rate), as well as the change in the sleep HR and HRV from the BL to the OL, were associated (*p* < 0.05) with a change in the 3000 m running time. In conclusion, the subjective recovery metrics were impaired by intensified training, while the sleep and nightly recovery metrics showed no consistent changes. However, there were substantial interindividual differences in nightly recovery, which were also associated with the training adaptations. Therefore, monitoring nightly recovery can help in recognizing individual responses to training and assist in optimizing training prescriptions.

## 1. Introduction

Wearable technology has been proposed as one of the major fitness trends for several years [1]. Wearables consist of a wide range of sensors that can be used in sports and exercise contexts for monitoring daily physical activity [2] or inactivity [3], internal and external training load metrics [4], and physiological recovery from training [5]. Examples of wearables include wrist-worn watches, rings, and clothing [6]. The measurement results are typically presented via product/brand-specific software that can provide raw data or data that have been analyzed with proprietary algorithms. In particular, recovery-related products provide estimates such as “readiness” [7] that are aimed at helping the consumers in their daily decision-making and adjusting exercise training appropriately [5].

In line with the growing popularity of wearables, sleep tracking has become more popular [8], also among athletic populations [6]. The gold standard method for sleep recording is polysomnography (PSG), which is based on the simultaneous recording of electroencephalogram, electromyogram, electrooculogram, and (optionally) a few other physiological signals [9]. By combining signals, sleep can be classified as REM or NREM, with the latter further divided into three stages of varying depth [10]. As a limitation, it is not feasible to implement the PSG method in day-to-day monitoring. Therefore, wearables can provide beneficial alternatives for objective sleep monitoring. At its simplest, the timing and duration of sleep can be estimated, and most of the consumer wearables do this accurately [6]. However, wearables perform worse in sleep stage detection [6], although by combining several signals the accuracy can be improved [11].

Beyond sleep tracking, wearables can also be used to monitor physiological functions such as breathing rate, skin temperature, oxygen saturation, and heart rate (HR) and heart rate variability (HRV) during sleep [12], which can provide further insights into recovery and training adaptation. Although these parameters can also be monitored during waking hours, sleep time can be considered a standardized state when there are minimal external disturbances, making it an ideal opportunity to observe meaningful changes in physiological regulations [13]. Regardless of certain changes in physiological functions (e.g., autonomic nervous system regulation) across sleep stages [14], the HR and HRV during sleep have been reported as reliable metrics to assess internal responses to training load [15].

In the sports context, sleep forms the foundation of effective recovery strategies, and its importance in the entire training process is currently well understood [16]. The potential consequences of insufficient sleep concern a wide range of domains, as it has been shown to negatively affect physical performance [17,18], skill-based performance [18], neurocognitive functions [17], and physical health [17]. Despite the detrimental effects, surprisingly few athletes reach their desired amount of sleep [19]. The physiological mechanisms between sleep and recovery are not yet fully understood, but sleep-time hormonal regulation has been suggested to be one potential contributor, at least for muscle repair [20]. In addition, an altered autonomic nervous system balance, e.g., decreased parasympathetic nervous system activity, could be associated with overreaching [21,22].

During periods of intensified training, the amount and quality of sleep could hypothetically become even more important. The effects of an increased training load on sleep have been examined in a few studies. While the results are slightly contradictory, it seems that the objective sleep parameters are more likely to deteriorate than the subjective perception of sleep quality [23]. For example, Hausswirth et al. [24] found that sleep (duration, efficiency, and immobility) was negatively affected by the overload period only in individuals who were classified as overreached, suggesting a potential link between sleep and training responses. While wearables have continued to evolve and are now able to measure multiple aspects of sleep, the challenge is that the actual relevance of several (proprietary) metrics that are provided is not often clear, especially in terms of individual training responses. Therefore, the purpose of this study was to examine the effects of intensified training on sleep and nightly recovery with a widely used commercial wearable and to analyze whether the observed responses were associated with the training-induced adaptations.

## 2. Materials and Methods

### 2.1. Participants

A total of 32 healthy recreational runners, training regularly 4–6 times per week, were recruited to participate. Their health status was screened with a custom questionnaire and a resting electrocardiography recording to exclude any symptoms or diseases (e.g., cardiovascular or musculoskeletal) that could have affected the participation. Eight participants withdrew due to scheduling conflicts (n = 2), unrelated health issues (n = 2), and leg injuries or soreness (n = 4). Thus, the final sample consisted of 24 participants (10 females). The baseline characteristics of the participants involved in the analyses are presented in Table 1. All of the participants gave their written consent, and the study protocol was approved by the ethics committee of the University of Jyväskylä.

### 2.2. Study Design

The study period consisted of three distinct phases in line with previous overreaching protocols [25,26,27]: a 3-week baseline training period (BL), a 2-week overload period (OL), and a 1-week recovery period (REC). The training load during the BL corresponded to the typical training volume of each participating individual (4–6 sessions and 4–7 h per week). During the OL, the planned increase in the training load was 80% compared to the BL as determined by the formula of Lucia et al. [28]. During the REC, the training load was decreased by 40% compared to the BL. The training load was defined based on the prescribed time in zone 1 (the HR below the first lactate threshold), zone 2 (the HR between the first and second lactate thresholds), and zone 3 (the HR above the second lactate threshold). While there exist several methods to quantify the training load [29], the current approach was chosen because it takes into account the varying levels of physiological strain across different intensity domains. Furthermore, since the training was prescribed based on lactate thresholds, this approach was the most practical one with regard to the training prescription. The appropriate changes in the training load were estimated based on previous study protocols that used a 2–3-week training period to induce functional overreaching [22,25,26,27]. The distance running performance was assessed before and after each period with a 3000 m running test. The 3000 m running test was chosen as an indicator of distance running performance due to its feasibility, reproducibility [30], and strong associations with the laboratory-based endurance performance indicators in recreational runners [31]. During the same test visit, the subjective sleep quality was assessed with the Athlete Sleep Screening Questionnaire (ASSQ) [32], and the general state of recovery was assessed with The Daily Analysis of Life Demands for Athletes (DALDA) questionnaire [33]. Sleep, nightly recovery, and subjective recovery based on recovery questions from the Polar Recovery Pro feature [34] were monitored on a daily basis throughout the study period. The protocol of the whole study has been described in more detail in a previously published article [35].

### 2.3. Sleep and Nightly Recovery

Sleep and nightly recovery were monitored with a wrist-worn Polar Vantage V2 (Polar Electro Oy, Kempele, Finland) every night during the study. The accuracy of the watch in sleep monitoring [36,37] and the recording of the HR and HRV [38] have been reported in previous validation studies. The participants were advised to attach the watch before going to sleep and to follow the manufacturer’s instructions on how to wear the watch properly. The data from the watch were synchronized to the Polar Flow service and then further transferred to the Coach4Pro software (Coach4Pro Oy, Espoo, Finland), from which the sleep-related parameters were exported. The sleep-related parameters provided by the watch and used in the current analyses are presented in Table 2.

### 2.4. Subjective Recovery Questions

Subjective recovery measures have been suggested to be sensitive in their responses to acute and chronic changes in the training load [41], and they can provide important supportive information beyond objective methods in monitoring athletes’ responses to training. Therefore, the present study also assessed sleep and recovery using several subjective assessments. In addition to the ASSQ and DALDA questionnaires, the participants’ sleep quality and subjective recovery were assessed daily via the Coach4Pro software. The sleep quality of the previous night was estimated as 1 = very poor, 2 = poor, 3 = normal, 4 = good, or 5 = very good. The perceived strain was estimated as 0 = normal, 1 = more than normal, or 2 = much more than normal, and the muscle soreness was estimated as 0 = normal, 1 = more than normal, or 2 = much more than normal.

### 2.5. Data Analyses

**Figure 1:**
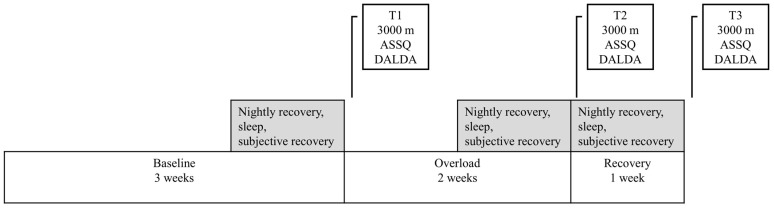
An illustration of the study period and analysis segments.

All of the sleep-related parameters and subjective recovery questions were analyzed as 7-day averages at the end of the baseline period (T1), after the overload period (T2), and after the recovery period (T3) (Figure 1). A similar approach has been used in previous studies assessing the effects of overload training [25,26,27], and weekly averages have been suggested to be methodologically superior compared to single-day results in the detection of functional overreaching [42]. In addition to the T1–T3 changes, all of the daily monitored parameters were analyzed as an average of the entire overload period. One participant was excluded from the HR- and HRV-based analyses due to abnormal data that prevented obtaining data from the watch recordings during the study period.

### 2.6. Statistical Analyses

The results are presented as the mean ± standard deviation (SD). The normality of the data was analyzed with the Shapiro–Wilk test. A repeated measures analysis of variance with the Bonferroni post hoc test was used to analyze the effects of time (T1, T2, T3) on the outcome variables. Since the results of the DALDA questionnaire were not normally distributed, the analyses were performed using the Friedman nonparametric test. The associations between the change from T1 to T2 or T1 to peak performance (either at T2 or T3) in the 3000 m running performance and nightly recovery metrics were analyzed with the Pearson correlation coefficient. The analyses of the changes in subjective recovery (strain and muscle soreness) were performed with the Spearman rank correlation since these results were not normally distributed. The effect sizes (ES) of the changes were examined with Cohen’s d. In the analyses, the standardizer was calculated using the SD of the difference. The magnitude of the ES was interpreted according to Cohen [43]: 0.2–0.49 = small, 0.5–0.79 = moderate, and at least 0.8 = large. All of the statistical analyses were performed with the IBM SPSS Statistics v.28 program (SPSS Inc, Chicago, IL, USA).

## 3. Results

### 3.1. Training Intervention

#### 3.1.1. Training Characteristics and Training Adaptations

**Figure 2:**
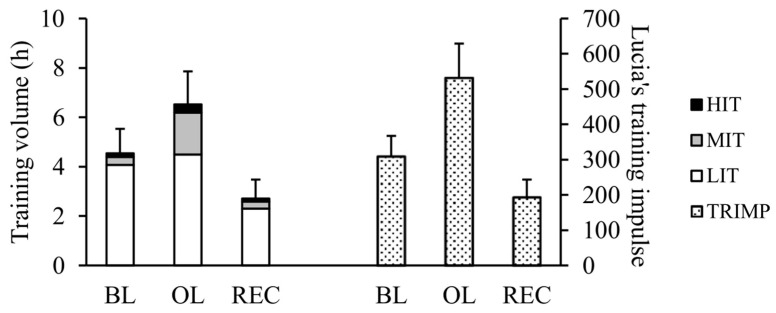
Mean (±SD) weekly training volume, training intensity distribution, and training load (TRIMP) during different training periods. HIT = high-intensity training; MIT = moderate-intensity training; LIT = low-intensity training; TRIMP = training impulse; BL = baseline training period; OL = overload training period; REC = recovery period.

The training volume, Lucia’s training impulse, and the running distance during the BL were 4.5 ± 1.0 h, 310 ± 58 a.u., and 43 ± 14 km, respectively. The training volume, training intensity distribution, and training load across the different training periods are demonstrated in Figure 2.

The 3000 m running time on T1 was, on average, 12:48 ± 1:50 min:s. The running time improved from T1 to T2 by −1.2 ± 1.7% (*p* = 0.006, ES = 0.71) and from T1 to T3 by −1.7 ± 1.2% (*p* = 0.002, ES = 0.81).

#### 3.1.2. Subjective Recovery

The number of negative responses on the DALDA B questionnaire increased (*p* = 0.02) from T1 (1.3 ± 1.3) to T2 (2.9 ± 2.7, ES = 0.66), but it returned to the baseline at T3 (1.2 ± 1.8, ES = 0.10). The perceived strain and muscle soreness increased (*p* < 0.001) from T1 (0.3 ± 0.3 and 0.2 ± 0.2) to T2 (0.7 ± 0.4, ES = 1.09 and 0.6 ± 0.5, ES = 1.09) and returned to the baseline at T3 (0.3 ± 0.4, ES = 0.08 and 0.3 ± 0.4, ES = 0.28).

#### 3.1.3. Intervention Effects on Sleep and Nightly Recovery Metrics

The ASSQ score did not change during the study period. In addition, the subjective sleep quality and objective nightly recovery metrics remained unchanged between T1 and T3 at the group level (Table 3).

#### 3.1.4. Watch Notifications During the Overload Period

**Figure 3:**
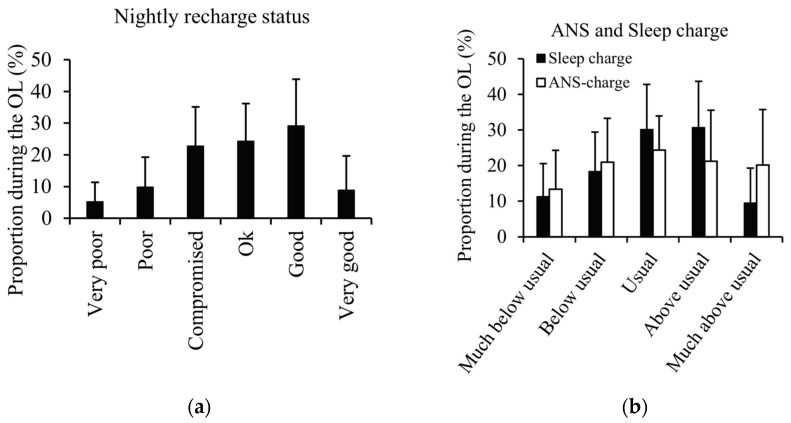
The proportion of the different types of notifications provided during the overload period in the (**a**) nightly recharge status and (**b**) ANS and sleep charge.

The proportion of the different types of notifications that were provided by the watch during the OL is presented in Figure 3. The negative nightly recharge status, sleep charge, and ANS charge notifications were provided on 38 ± 18%, 30 ± 13%, and 34 ± 17% of the nights, respectively.

### 3.2. Associations Between Nightly Recovery Metrics and Running Performance

**Figure 4:**
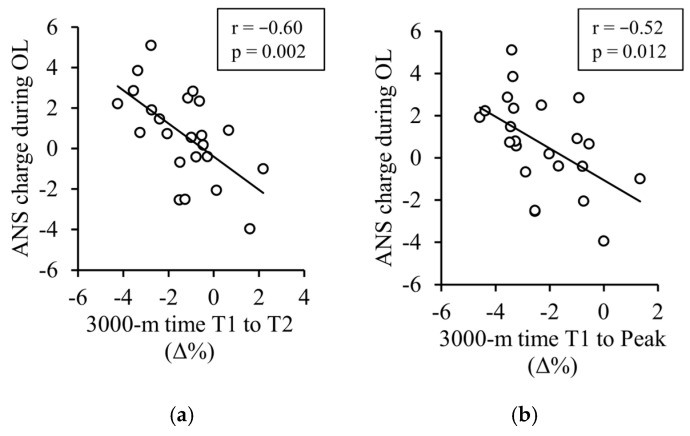
The associations between the average ANS charge (−10 to 10) during the entire OL and the 3000 m training adaptations.

Table 4 presents the associations between the absolute results of the nightly recovery metrics during the OL, the changes in the nightly recovery metrics from T1 to T2, and the training adaptations. The greatest correlations were found for the average ANS charge during the entire OL (Figure 4).

## 4. Discussion

One of the main discoveries of this study was that the sleep and nightly recovery metrics were not systematically affected by short-term intensified training, although the subjective recovery was impaired. Nevertheless, there were substantial interindividual differences in the sleep-related responses, and the objectively measured nightly recovery was associated with the training adaptations, supporting its relevance in recovery monitoring.

Previous studies have been somewhat contradictory regarding the effects of overreaching periods on sleep quality. Based on a recent meta-analysis, subjective sleep quality is less affected by overreaching, while objective sleep (sleep efficiency) is more likely to become impaired [23]. However, as Walsh et al. [16] have highlighted, such differences are likely to be small in their magnitude. The potential reasons for sleep deterioration due to intensified training or overreaching are related to several mechanisms. For example, altered immune function due to physical [16] and mental stress [44] can impair sleep quality. Changes in mood/anxiety are considered potential symptoms of overreaching, and these by themselves can have negative effects on sleep [16]. Hausswirth et al. [24] also speculated that increased muscle fatigue/soreness could influence sleep efficiency. When considering the current results (the lack of systematic changes in sleep or nightly recovery metrics) during both increased and decreased training loads, it is important to acknowledge that the duration of the OL was relatively short (2 weeks), and only eight participants were diagnosed as overreached [35]. On the other hand, changes in the DALDA B responses, perceived strain, and perceived muscle soreness confirmed that the current OL period was demanding compared to the normal training routines of recreational runners.

Another key finding in the current study was that the variables related to the autonomic nervous system (ANS) regulation seemed to be more clearly associated with the training adaptations compared to the parameters related to sleep itself (e.g., duration, efficiency, perceived quality). The associations between endurance-training adaptations and changes in HR/HRV have also been reported previously [45,46,47] but mainly after regular training periods. In terms of overload periods, contrasting results have even been reported for resting HRV measured in the morning, suggesting that increased HRV could also be associated with negative training adaptations and functional overreaching [25,26,42]. It is possible that the “parasympathetic hyperactivity” phenomenon does not concern sleep-time ANS regulation, as it has not been reported in studies using nocturnal recordings [42]. Another explanation might relate to the baseline fitness level and absolute training volume of the participants, both of which have been greater in many studies reporting parasympathetic hyperactivity [42].

While multiple recovery processes take place during sleep [16,48], disturbances in these mechanisms could lead to compromised training outcomes. Since the ANS mediates many physiological processes that relate to the restoration of body homeostasis [49], it would be logical that abnormal HR and HRV responses could be a sign of delayed recovery processes and/or a negative state of recovery (overreaching). Some studies have indeed reported an altered sleep-time HRV regulation during slow wave sleep [22] or during a 6 h recording [21] due to overreaching, thus supporting this hypothesis. Although there seems to be a link between training adaptations and nocturnal parasympathetic nervous system activity, it is challenging to estimate whether the diminished parasympathetic nervous system activity during sleep contributes, by itself, to poorer training tolerance or whether it is a secondary outcome of an impaired state of recovery. The relevance of maintaining a sufficient level of parasympathetic nervous system activity at rest has been examined in training interventions that have adjusted the training load of participants based on their resting HRV status [50]. Although this type of approach seems to be beneficial for endurance performance, further studies are needed to better understand the actual mechanisms behind resting ANS regulation and training adaptations.

In addition to sleep and nightly recovery responses, another key aspect of this study was to examine how useful the feedback of the watch could be in preventing an excessive training load. Although “raw data” are important, especially for research purposes, actual consumers may not have the skills or time to interpret these results. Therefore, the interpretations and subsequent actions can often rely more on algorithm-based metrics, such as “readiness scores” [7]. It is known that many recovery-related parameters are highly individual, which is why it is important to define the normal range that would reveal whether a change in a certain parameter could be considered worthwhile [51]. This type of approach was also applied to the current nightly recovery metrics (e.g., the ANS charge and sleep charge). Interestingly, a poorer average ANS charge and a greater proportion of negative notifications of nightly recharge during the OL were associated with smaller training adaptations. Thus, modifying the training prescription according to this type of feedback could be beneficial. For example, the planned training load for the day can be decreased, or the start of an intensive training block can be delayed, if negative patterns are observed in the nightly recovery metrics. Although even one night of compromised sleep can affect physical performance [52], it is generally recommended that the interpretation of resting HRV should be based on averages over several days [53] to improve the result’s reliability. On the other hand, day-to-day variation in nocturnal recordings is smaller than in the morning recordings [54], which may allow utilizing shorter assessment windows. An interesting finding about feedback on sleep quality is its potential placebo effects, particularly on cognitive functions [55]. Therefore, it would be very important to understand the limitations of different types of metrics and focus only on those that provide reliable and valid information.

The concept of overall sleep quality is broad, and it can be considered a combination of subjective and objective viewpoints [56]. Regarding the comparison between objective and subjective recovery/training load markers in general, Saw et al. [41] have suggested that subjective markers are more sensitive in their responses to training load. In the current study, the subjective sleep quality remained unchanged, but other subjective recovery markers (DALDA B responses, strain, muscle soreness) became impaired. In the monitoring of athletic training, it is relevant to note that impaired subjective recovery is not automatically associated with maladaptation to training, since it could also reflect the increase in training load by itself. Therefore, the magnitude of a “worthwhile” negative change in subjective markers is likely larger than is required for objective markers in the prediction of overreaching [35]. When monitoring recovery, it is important to distinguish the training load from the internal response it induces. The current study demonstrated how significant short-term increases in the training load can be tolerable for recreational runners. It is known that several factors besides the training itself, such as baseline fitness, nutrition status, and mental stressors, can contribute to the recovery-adaptation processes and interindividual variability in training tolerances [57]. Therefore, objective recovery markers can help confirm that the training load is not excessive at a given moment, despite possibly impaired subjective recovery. The information can also be applied to individual training prescriptions [50,51]. For instance, the training progression can be adjusted based on nightly recovery. With this kind of individualized approach, it could be possible to reduce the differences between individuals and increase the likelihood of positive training responses [50,51].

The current study focused on recreational runners; thus, the results cannot be extrapolated to elite athletes who have greater absolute training loads and potentially diverse external stressors. Factors such as diet, mental stress, and free-time activities were not controlled, and it should be acknowledged that these can influence sleep- and recovery-related results. The current study focused on a single commercially available wearable, and the metrics that were analyzed were limited to those provided by the watch. Thus, the results cannot be directly applied to other wearables. Moreover, there may be some other sleep-related parameters (e.g., sleep onset latency or sleep fragmentation metrics) that may have a distinct pattern compared to the currently assessed metrics. Therefore, it is suggested that various potential sleep-related metrics and parameters be examined in greater detail in the future, considering variations in training load and recovery states.

## 5. Conclusions

Daily sleep monitoring was conducted in the present study, and training adaptations were examined in connection with sleep-related parameters. These results provide practical insights for the monitoring of athletic training. In conclusion, the subjective recovery metrics were impaired by intensified training, while the sleep and nightly recovery metrics showed no consistent changes. It is noteworthy that there were substantial interindividual differences in nightly recovery, which were also associated with the training adaptations. Therefore, monitoring nightly recovery can help recognize individual responses to training and assist in optimizing the training prescriptions for athletes.

## Figures and Tables

**Table 1:** The baseline characteristics of the participants.

	Participants (n = 24)
Sex (females/males)	14/10
Age (yrs)	38.6 ± 6.5
Height (cm)	173.2 ± 9.7
Body mass (kg)	72.6 ± 14.0
3000 m time (s)	13:03 ± 1:56

**Table 2:** The sleep and nightly recovery metrics that were recorded with the Polar Vantage V2 (Polar Electro Oy, Kempele, Finland). More detailed descriptions of the proprietary features can be found in the white papers [39,40] provided by the manufacturer.

	Description
Sleep time	Time between sleep onset and offset (h).
Actual sleep	Time scored asleep between sleep onset and offset/sleep time x 100 (%).
Sleep continuity	An estimate of how continuous the sleep was on a scale of 1.0–5.0, where 5.0 reflects uninterrupted sleep. The lower the value the more fragmented the sleep was.
Sleep score	A summary parameter that combines sleep time and quality on a scale of 1–100.
Sleep charge	Sleep score compared to an individual’s usual level from the past 28 days.
Sleep charge notification	Textual feedback on sleep charge on a 5-item scale: much below usual, below usual, usual, above usual, much above usual.
HR	Average heart rate from a 4 h period starting at 30 min after detected sleep onset (bpm).
RMSSD	Average root mean square of successive differences from a 4 h period starting at 30 min after detected sleep onset (ms). RMSSD is considered as a non-invasive method to assess cardiac parasympathetic nervous system activity.
Breathing rate	Average breathing rate as breaths per minute during a 4 h period starting at 30 min after detected sleep onset (bpm).
ANS charge	ANS stands for autonomic nervous system. ANS charge combines HR, RMSSD, and breathing rate. It is formed comparing the last night’s values to an individual’s usual level from the past 28 days. The scale is from −10.0 to +10.0.

**Table 3:** The subjective sleep quality and nightly recovery metrics before the overload period (T1), after the overload period (T2), and after the recovery period (T3). The results represent 7-day average values on the test days. The effect size (ES) results are presented with the 95% confidence intervals in the brackets.

	T1	T2	T3	ES T1–T2	ES T2–T3	ES T1–T3
ASSQ	4.9 ± 2.1	4.5 ± 1.8	4.8 ± 2.3	−0.29 (−0.70; 0.12)	0.15 (−0.26; 0.55)	−0.06 (−0.46; 0.34)
Subjective sleep quality (1–5)	3.3 ± 0.4	3.2 ± 0.5	3.4 ± 0.5	−0.16 (−0.56; 0.24)	0.44 (0.02; 0.86)	0.16 (−0.24; 0.57)
Sleep continuity (1–5)	3.2 ± 0.6	3.1 ± 0.6	3.0 ± 0.7	−0.18 (−0.58; 0.23)	−0.29 (−0.69; 0.12)	−0.52 (−0.94; −0.08)
Sleep score (0–100)	75.9 ± 7.4	75.6 ± 7.8	75.2 ± 9.0	−0.08 (−0.48; 0.32)	−0.08 (−0.48; 0.32)	−0.15 (−0.55; 0.25)
Sleep time (h)	7.2 ± 0.8	7.1 ± 0.8	7.2 ± 0.2	−0.20 (−0.60; 0.20)	0.21 (−0.20; 0.61)	0.00 (−0.40; 0.40)
Actual sleep (%)	93.7 ± 1.6	93.6 ± 1.9	93.1 ± 3.5	−0.06 (−0.46; 0.34)	−0.25 (−0.65; 0.16)	−0.26 (−0.66; 0.15)
HR (bpm)	51.4 ± 6.9	51.7 ± 7.7	50.8 ± 8.6	0.10 (−0.31; 0.51)	−0.24 (−0.65; 0.18)	−0.16 (−0.57; 0.25)
RMSSD (ms)	70 ± 22	71 ± 24	71 ± 23	0.10 (−0.31; 0.51)	−0.01 (−0.42; 0.40)	0.10 (−0.32; 0.50)
Breathing rate (bpm)	13.7 ± 1.0	13.7 ± 0.9	13.7 ± 0.9	−0.26 (−0.67; 0.16)	0.00 (−0.41; 0.41)	−0.28 (−0.69; 0.14)

ES = effect size, HR = heart rate, RMSSD = root mean square of successive differences.

**Table 4:** Pearson correlation coefficients between subjective recovery, nightly recovery metrics, and changes in 3000 m running time.

	3000 m Change T1 to T2	3000 m Change T1 to Peak
Absolute OL results		
Perceived strain (0–2)	0.24	0.27
Perceived muscle soreness (0–2)	0.14	0.20
Subjective sleep quality (1–5)	−0.03	−0.11
Sleep time (h)	0.20	0.22
Sleep score (1–100)	0.09	0.10
Actual sleep (%)	−0.03	−0.03
Sleep continuity (1–5)	−0.05	0.03
ANS charge (−10 to 10)	−0.60 **	−0.52 *
Sleep charge (−10 to 10)	0.21	0.01
HR (bpm)	0.09	0.29
RMSSD (ms)	0.01	−0.04
Breathing rate (rpm)	0.39	0.52*
Change from T1 to T2		
Perceived strain (0–2)	0.23	0.14
Perceived muscle soreness (0–2)	0.50 *^a^	0.32 ^a^
Subjective sleep quality (1–5)	−0.24	−0.15
Sleep time (h)	0.14	0.16
Sleep score (1–100)	−0.11	−0.05
Actual sleep (%)	−0.30	−0.23
Sleep continuity (1–5)	−0.34	−0.18
HR (bpm)	0.44 *	0.49 *
RMSSD (ms)	−0.48 *	−0.45 *
Breathing rate (bpm)	0.30	0.15
Proportion of negative notifications during the OL		
Nightly recharge status	0.48 *	0.36
ANS charge	0.37	0.25
Sleep charge	0.38	0.24

HR = heart rate, RMSSD = root mean square of successive differences. ^a^ Analyzed with Spearman rank correlation. * *p* < 0.05, ** *p* < 0.01.

## Data Availability

Data are available upon reasonable request from the corresponding author.
